# Combination therapy of anti-cancer bioactive peptide with Cisplatin decreases chemotherapy dosing and toxicity to improve the quality of life in xenograft nude mice bearing human gastric cancer

**DOI:** 10.1186/2045-3701-4-7

**Published:** 2014-02-10

**Authors:** Xiulan Su, Chao Dong, Jialing Zhang, Liya Su, Xuemei Wang, Hongwei Cui, Zhong Chen

**Affiliations:** 1 Clinical Medicine Research Center of The Affiliated Hospital, Inner Mongolia Medical University, No 1 Tongdao North Street, Huimin District, Hohhot, Inner Mongolia 010050, China; 2 PET-CT Center of The Affiliated Hospital, Inner Mongolia Medical University, No 1 Tongdao North Street, Huimin District, Hohhot, Inner Mongolia 010051, China; 3 Tumor Biology Section, Head and Neck Surgery Branch, National Institute on Deafness and Other Communication Disorders, National Institutes of Health, Bethesda, Maryland 20892-1419, USA

**Keywords:** Anti-cancer bioactive peptide (ACBP), Cisplatin, Tumor growth, Quality of life, Gastric cancer

## Abstract

**Background:**

A great challenge of cancer chemotherapy is to eliminate cancer cells and concurrently maintain the quality of life (QOL) for cancer patients. Previously, we identified a novel anti-cancer bioactive peptide (ACBP), a peptide induced in goat spleen or liver following immunization with human gastric cancer protein extract. ACBP alone exhibited anti-tumor activity without measurable side effects. Thus, we hypothesize that ACBP and combined chemotherapy could improve the efficacy of treatment and lead to a better QOL.

**Results:**

In this study, ACBP was isolated and purified from immunized goat liver, and designated as ACBP-L. The anti-tumor activity was investigated in a previously untested human gastric cancer MGC-803 cell line and tumor model. ACBP-L inhibited cell proliferation *in vitro* in a dose and time dependent manner, titrated by MTT assay. The effect of ACBP-L on cell morphology was observed through light and scanning electron microscopy. *In vivo* ACBP-L alone significantly inhibited MGC-803 tumor growth in a xenograft nude mouse model without measurable side effects. Treatment with the full dosage of Cisplatin alone (5 mg/kg every 5 days) strongly suppressed tumor growth. However, the QOL in these mice had been significantly affected when measured by food intakes and body weight. The combinatory regiment of ACBP-L with a fewer doses of Cisplatin (5 mg/kg every 10 days) resulted in a similar anti-tumor activity with improved QOL. ^18^F-FDG PET/CT scan was used to examine the biological activity in tumors of live animals and indicated the consistent treatment effects. The tumor tissues were harvested after treatment, and ACBP-L and Cisplatin treatment suppressed Bcl-2, and induced Bax, Caspase 3, and Caspase 8 molecules as detected by RT-PCR and immunohistochemistry. The combinatory regiment induced stronger Bax and Caspase 8 protein expression.

**Conclusion:**

Our current finding in this gastric cancer xenograft animal model demonstrated that ACBP-L could lower Cisplatin dose to achieve a similar anti-tumor efficacy as the higher dose of Cisplatin alone, through enhanced modulation of apoptotic molecules. This newly developed combination regiment improved QOL in tumor bearing hosts, which could lead to clinical investigation for the new strategy of combination therapy.

## Background

Cancer is a genetic disease that is developed due to accumulated multiple genetic defects along human life span. The heterogenous natures of genetic and malignant phenotypes within each type of cancer create a great challenge for cancer diagnosis and treatment [1]. At present the time, a high percentage of cancer patients are incurable, especially for solid tumors in late stage [2]. So, the quality of life (QOL) is particularly important to patients with advanced cancer, because most of them are symptomatic when diagnosed [3,4]. In addition, repeated utilization of chemotherapy agent nonspecifically kills proliferating cells, which leads to significantly toxic side effects and decreased patient QOL [5]. Such nonspecific therapeutic strategy induces chemo-resistance, which creates a situation of either further increasing chemotherapy dosage with more severe toxicity or making patients intolerable for treatment. Neither is a best therapeutic strategy for cancer patients. Therefore, when treating cancer patients at the intermediate or advanced stage, the evaluation of patient outcomes should not just focus on the elimination of tumor burden at the expense of cancer patient QOL.

Cisplatin is one of the first line chemotherapy agents for treating advanced gastric cancer [6]. Previously, many clinical trials have been conducted to find the best combinatory regiment of Cisplatin with other chemotherapy agents, such as Docetaxel and Fluorouracil [7]. However, cancer patient QOL was ignored in many of the clinical trials because the traditional way to evaluate the efficacy of cancer therapy usually only relies on the index of cure rate and survival rate. As recent bio-psyco-social models have been developed to evaluate total cancer patent condition as a whole biological system, more attention has been drawn to the QOL of cancer patients during treatment [8]. Currently, QOL is one of several important indicators used to evaluate the efficacy of treatment, which is not only based on clinical objective indexes as evaluation standard, but also emphasizes subjective conditions of cancer patients [9]. For example, prospectively assessed QOL (even after completion of protocol treatment) was proposed and performed as one of the secondary end points of the phase III trial of combined therapy of Docetaxel plus Cisplatin and Fluorouracil for advanced gastric or gastroesophageal adenocarcinoma [10].

Anti-cancer treatments using biologically active materials, including bioactive peptides, have recently been identified with potent anti-cancer activity and lack of side effects [11-13]. Bioactive peptides exist naturally in living beings such as animals, plants, and microorganisms. In addition, these peptides can be produced through artificial modification of biological materials, such as proteolysis of tissues and serum from animals or plants; or synthetic methods, such as chemical synthesis or biological engineering. The naturally existing bioactive peptides play a crucial role in regulating biological activities, including molecular recognition, signaling transduction, cell proliferation, and differentiation. Previously, we identified anti-cancer bioactive peptide (ACBP), which is such a naturally existing peptide [14]. ACBP is a mixture of several polypeptides with a molecular weight of about 8 kD, and isolated and purified from goat spleens or livers after immunization with human gastric cancer protein extracts. ACBP was analyzed using high performance capillary electrophoresis (HPCE) and matrix-assisted laser desorption-ionization time-of-flight mass spectrometry (MALDI-TOF-MS) [15,16]. ACBP exhibited multiple biological activities, including anti-tumor activity *in vitro* and *in vivo*[14]. In addition, acute and chronic toxicological tests in mice and rats showed no measurable toxicities or side effects interfering with normal physiological functions and enzyme metabolism activities [17,18].

Based on the low toxicity of ACBP, we hypothesized that ACBP could potentiate chemotherapy agent, enhance the efficacy of treatment, and lower the chemotherapy dosage to decrease drug induced side effects and toxicity. However, the combinatory effects of ACBP and chemotherapy have never been investigated in animal tumor models. In this study, we used a combined regiment of ACBP-L (from liver) with a lower dosing Cisplatin, which could reach the same anti-tumor efficacy as the higher dosing Cisplatin alone in the xenograft nude mouse model bearing human gastric cancer MGC-803. Decreased chemotherapy dosage leads to improved QOL of tumor-bearing nude mice. The molecular mechanisms of the combined therapy could be regulated through the modulation of apoptotic molecules, such as BAX, Bcl-2, Casapse 3, and Caspase 8. Our study suggests that the combination of ACBP-L and chemotherapy could be a new anti-cancer strategy, which is capable of concurrently suppressing tumor growth and improving host QOL.

## Results

### ACBP-L exhibited anti-tumor activity against MGC-803 cancer cells in a dose and time-dependent manner *in vitro*

**Figure 1:**
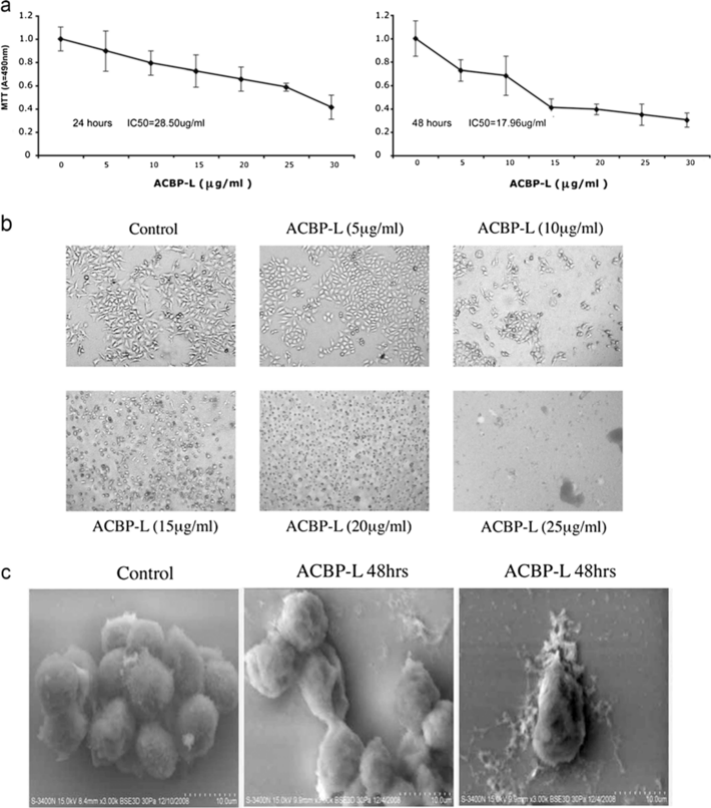
**ACBP-L exhibits anti-tumor activity against MGC-803 cancer cells in a dose and time-dependent manner *in vitro. *(a)** ACBP-L suppressed MGC-803 gastric cancer cell proliferation at a dose-dependent manner by MTT assay measured at the absorbance of 490 nm (A = 490 nm) during 24 hr (left) and 48 hr (right) time points. The samples were measured in triplicates and the data were presented as mean ± standard deviation (SD). The median concentration of inhibition (IC_50_) at 24 hr is 28.50 μg/ml, and at 48 hr is 17.96 μg/ml. **(b)** ACBP-L inhibited cell growth and induced cell apoptosis in a dose dependent manner observed under light microscopy (400X). **(c)** Scanning electron microscopy showed cell membrane damage after 48 hr ACBP-L treatment (shown in two different fields). Scale indicates 10 μM.

To determine the effect of ACBP-L on cell proliferation, MGC-803 cancer cells were treated with increasing concentrations of ACBP-L for 24 hrs and 48 hrs. Increased ACBP-L (5.0-30.0 μg/mL) inhibited cell proliferation in a dose dependent manner measured by MTT assay (Figure 1a). The survival of MGC-803 cancer cells was decreased by approximately 10.3%, 20.7%, 27.6%, 34.5%, 41.4%, and 58.6% after a 24 hr exposure to 5, 10, 15, 20, 25, or 30 μg/ml ACBP-L, respectively. The median concentration of inhibition (IC_50_) at 24 hrs was 28.50 μg/ml. In addition, ACBP-L anti-proliferative effect on MGC-803 cells was persistent and increased with prolonged treating time. The inhibitory rates at 48 hrs were: 27.3%, 31.8%, 59.1%, 60.6%, 65.2%, and 69.7%, respectively, and IC_50_ = 17.96 μg/ml. We compared the inhibitory rates at the different time point and found that, a 48-hour treatment of MGC-803 cancer cell with the 25 μg/ml concentration of ACBP-L resulted in a 65.2% decrease in cell viability, compared with a 41.4% decrease with 24-hour exposure to the same concentration of ACBP-L (Figure 1a).

The cell morphology after different doses of ACBP-L treatment was observed (Figure 1b). The cell morphology resembled cell growth inhibition and an induction of cell apoptosis. At the lower dose (10 μg/ml), fewer cells were observed in the culture, and the remaining cells exhibited the morphology of bleb, loss of cell membrane asymmetry, and detachment. At the higher concentration (15 μg/ml), cell shrinkage and nuclear condensation were more apparent. In addition, under the treatment of the highest concentrations of the two doses, cells completely lost membrane and exhibited condensed nucleus, or only cell debris was left (Figure 1b). Scanning electron microscope revealed a consistent morphology that cell membrane was damaged and lost asymmetry (Figure 1c).

### ACBP-L potentiated the low dose Cisplatin treatment to suppress gastric tumor growth in a xenograft tumor model

**Figure 2:**
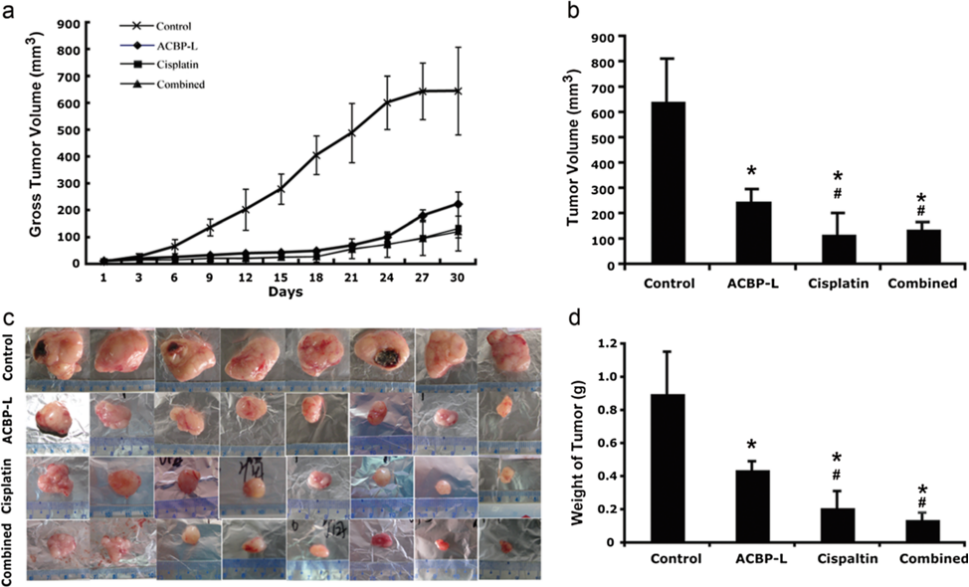
**ACBP-L and low dose Cisplatin combined treatment suppresses gastric tumor growth in the xenograft tumor model.** ACBP-L suppresses tumor growth *in vivo*. The *in vivo* tumor growth experiment was established by subcutaneous injection of 2×10^7^ MGC-803 gastric cancer cells. After tumors were palpable and when the sizes of tumors were about ~10 mm^3^, the tumor bearing mice were randomized with eight mice (n = 8) each into four groups: control with daily intraperitoneal injection of saline, daily injection of ACBP-L (7 μg/mouse) alone, Cisplatin alone (5 mg/kg every five days for 4 times, given the drug at day 6, 11, 16, 21), and Cisplatin (5 mg/kg, twice giving at day 6 and 16) plus daily ACBP-L. The tumor volumes were measured every three days **(a)**. The final tumor volumes were calculated before the end of experiments **(b)**. The data were calculated and presented as mean ± standard deviation (SD). Statistical significance was determined by Student t-test (statistical difference was indicated as p < 0.05, when compared with the control group (*), or compared with ACBP-L treated alone group (#). **(c and d)** Tumor weight was measured after harvest, and the data were calculated and presented as mean ± standard deviation (SD). Statistical significance (p < 0.05) was determined by Student t-test when compared with the control (*) and ACBP-L treated alone group (#).

A xenograft nude mouse model was established with subcutaneous inoculation of human gastric MGC-803 cancer cells. The tumor growth rate was measured and calculated at each time point, and the statistical significance of the tumor growth rate was examined (Figure 2a). In the control group, tumor volume increased significantly shown by each measurement, indicating an aggressive malignant phenotype with a fast tumor growth rate. The anti-tumor activity of ACBP-L was tested by daily injection of 7 μg/mouse, and compared with traditional regiment of Cisplatin alone at 5 mg/kg four times every five days. The regiments are the essential dosages with anti-tumor activity identified from pilot experiments (data not shown). To test if ACBP-L could potentiate Cisplatin anti-tumor effects, we decreased dose of Cisplatin to 5 mg/kg for every 10 days, with a total of two injections, and combined with ACBP-L (7 μg/mouse for daily injection). When compared the dynamic growth rate of the control and the three treated groups, the tumor growth rates were statistically different after treatment at day 7. At the end of treatment, ACBP-L significantly inhibited tumor growth by 61.3%, Cisplatin at the higher dose exhibited strongest anti-tumor activity with an inhibitory rate of 81.6% (Figure 2b, Student t test, p < 0.05). When ACBP-L was combined with low dose of Cisplatin, the treatment suppressed tumor growth at the similar rate as the high dose of Cisplatin alone (Figure 2a). At the end of the treatment, the combinatory treatment decreased tumor growth by 78.3% (Figure 2b, Student t test, p < 0.05). After harvesting tumors at the end of the experiment, the tumor weights of four experimental groups were examined and compared: control, 0.90 ± 0.25 g; ACBP-L, 0.44 ± 0.05 g; Cisplatin, 0.21 ± 0.10 g; combination, 0.14 ± 0.04 g (Figure 2c, d). The inhibition rate was ACBP-L, 51.1%; Cisplatin, 76.7%; combined therapy 84.4%. The statistical significance of tumor weights resembled those of tumor volumes (Figure 2b, d). There are statistical differences observed when comparing each treated groups with the control, however, high dose Cisplatin alone or combinatory treatment with low dose of Cisplatin exhibited the strongest anti-tumor effect (Student t test, p < 0.05).

### ACBP-L alone or in combination with the low dose Ciaplatin improve quality of life in the xenograft tumor model

**Figure 3:**
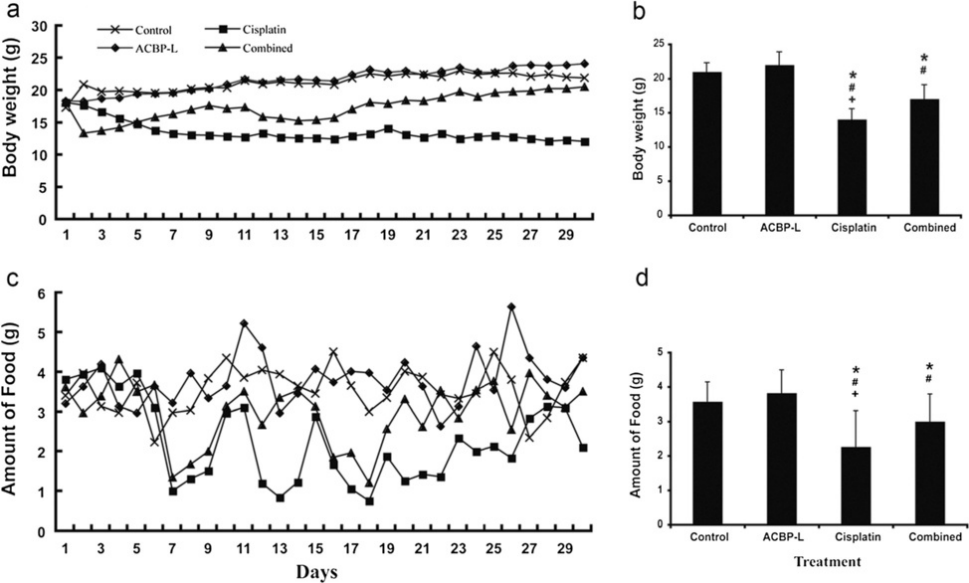
**ACBP-L and low dose Cisplatin combined treatment improves quality of life in the xenograft tumor model. (a)** Body weight of xenografted nude mice (n = 8) was measured every two days for thirty days, and means of the body weight of each group were presented. **(b)** Body weight was presented by mean ± (SD). Statistical significance were determined by Student t-test when compared with the control (*), ACBP-L (#), and combined treatment (+), p < 0.05. **(c)** Food intake by tumor bearing mice was measured every two days, and the means of each group were presented. **(d)** Data of food intake were presented as mean ± SD. Statistical significance was determined by Student t-test when compared with the control (*), ACBP-L (#), and combined treatment (+), p < 0.05.

When we examined the ACBP-L and Cisplatin anti-tumor activity *in vivo*, we also observed the quality of life (QOL) of tumor bearing animals. The QOL of ACBP-L and combinatory treated groups were significantly improved over that of the high dose Cisplatin group, indicated by body weight and food intake (Figure 3). The mice in the ACBP-L or combinatory treated groups were more active, had good appetite, and their appearance and body weight were close to that of a normal mouse. The mice in the group with high dosage of Cisplatin exhibited strong gastrointestinal toxicity (diarrhea), systemic toxicity (piloerection and lethargy), and a consistent decline in body weight (Figure 3a). At the end of experiment, there was no body weight loss in ACBP-L treatment when compared to control. The body weight of the high dose Cisplatin group was the lowest, and the body weight of the combinatory treated group was slightly lower than the control and ACBP-L treated groups (Student t test, p < 0.05, Figure 3b).

QOL was also examined by daily food intake of the experimental animals (Figure 3c). There were three large decreases of food intake in the Cisplatin alone group after the mice were given the 1^st^, 2^nd^, 3^rd^ doses of Cisplatin at day 6, 11, and 16. In the combinatory treated group, there were two decreased food intake corresponding to the Cisplatin dosing at day 6 and 16. At the end of the experiment, daily food intake by mice in the Cisplatin group was significantly lower than that of the other three groups (Figure 3d, Student t test p < 0.05).

### High dose of Cisplatin alone decreased spleen weight in the xenograft tumor model

**Figure 4:**
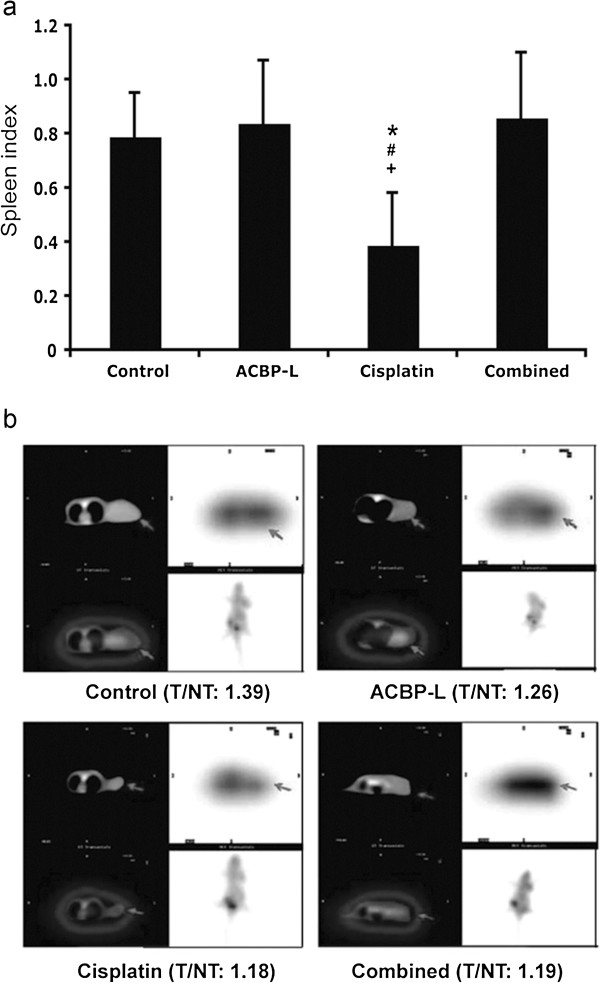
**ACBP-L or Cisplatin alone, or combined treatment suppresses metabolic activities in tumor baring animals and in live tumor cells *in vivo*. (a)** Spleen weight of each mouse (n = 8) was measured after euthanasia. Mouse spleen index was calculated by mouse spleen weight (mg) divided by the mouse body weight (g). Data are presented as mean ± SD from eight mice in each group. Statistical significance was determined by Student t-test when compared with the control (*), ACBP-L (#), and combined treatment (+), p < 0.05. **(b) **^18^F-FDG PET/CT fuse imaging (cross section) from a representative nude mouse of each group is presented (CT image, left; PET image, right). Radioactivity uptake represents the biological and metabolic activities of tumors (Target, T, black arrows) were compared with spine counts (Non-Target, NT) within the identical section, as the T/NT ratio. The T/NT ratio is significantly decreased in treated groups when compared with the control.

The spleen index was measured as an indication of systemic toxicity induced by Cisplatin (Figure 4a). There was a significant decrease of spleen index in the high dose of Cisplatin group when compared with the other three experimental groups, while the combination treatment group did not show significant decrease of spleen index, indicating less toxicity. However, no significant decrease of the liver weight was observed in any of the experimental groups (data not shown).

### ACBP-L and Cisplatin alone, or in combination, suppressed biological and metabolic activities in live tumor cells by PET-CT image

Viability and metabolic activity of xenograft tumors from four experimental groups were evaluated by ^18^F-FDG PET/CT imaging (Figure 4b). PET with ^18^F-FDG is a noninvasive approach for determination of the glycolytic status, and enhanced glycolysis is one of the most important characteristics of energy metabolism in cancer cells. The higher the ratio of radioactivity uptake in tumor versus normal area (Target/Non-target) indicates the stronger glycolysis in tumor cells, suggesting more energetic and aggressive tumor cell status. In this study, we observed a significant difference in radioactivity uptake when comparing the Target/Non-target ratio of tumor/spine (T/NT:1.39) in the control group with mice from three treated groups, ACBP-L (T/NT:1.26), Cisplatin (T/NT:1.18), and combined therapy (T/NT:1.19). Our data suggested that all three treatment regiments decreased the viability and metabolic activity of live tumor cells, where comparable inhibitory effects were observed in higher dosing Cisplatin treatment alone as the combined therapy of ACBP-L with lower dosing of Cisplatin (Figure 4b).

### ACBP-L and Cisplatin alone, or in combination, induced molecules promoting cell apoptosis

**Figure 5:**
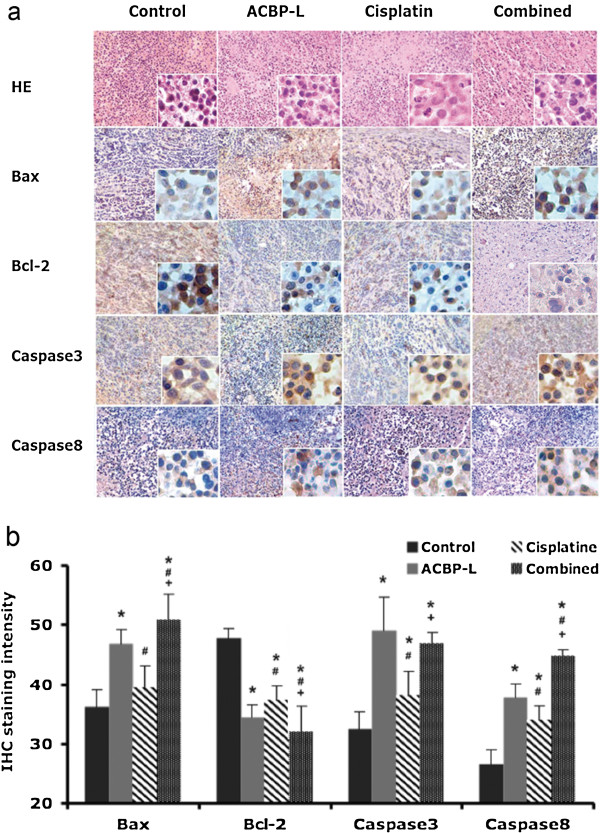
**ACBP-L or Cisplatin alone, or combined treatment induces molecules promoting cell apoptosis *in vivo. *(a)** Paraffin embedded tumor specimens (n = 8/each group) were stained with HE and immunohistochemistry of Bax, Bcl-2, Caspase 3, and Caspase 8, (low magnification 40X, high magnification 400X). **(b)** The quantitation of IHC scores were analyzed, the statistical significance was determined by Student t-test (p < 0.050), and data are presented as compared with control (*), ACBP-L (#), and Cisplatin treated group (+).

We hypothesized that the anti-tumor activity of ACBP-L could be mediated through promotion of tumor cell apoptosis. Pathological analysis after H&E staining of harvested tumors showed more cells with apoptotic features in all treated groups (Figure 5). Immunohistochemical staining of proteins involved in apoptosis were performed and quantified, that significant differences in Bax, Bcl-2, Caspase 3, and Caspase 8 expression were observed between the experimental groups with controls (Figure 5). Stronger Bax staining was observed in ACBP-L alone and in the combined treatment groups when compared with the control. Decreased Bcl-2 expression was observed in all treated groups. Induction of Caspase 3 and Casapse 8 were observed in all treated groups, and a stronger response was observed in the ACBP-L alone or the combinatory group (Student t test, p < 0.05).

**Figure 6:**
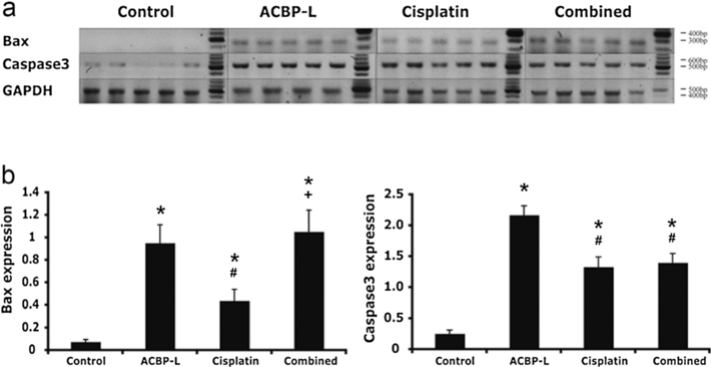
**Combined ACBP-L and Cisplatin treatment strongly induces anti-tumor activity in tumors Specimens. (a)** Semi-quantitative RT-PCR was performed to detect gene expression of Bax and Caspase 3 in tumor specimens (n = 5). RT-PCR products were run on 2% agarose gel, and gel images were shown with the indicated molecular weight. **(b)** The ratio of Bax and Caspase 3 gene expression were calculated and presented as mean ± standard deviation (SD), and the significant difference was examined by Student t-test (p < 0.050), and presented as compared with control (*), ACBP-L (#), and Cisplatin (+).

To further evaluate the molecular regulatory mechanism induced by ACBP-L and Cisplatin alone, or by the combinatory treatment, we analyzed the important apoptotic genes, such as Bax and Caspase 3 in tissue specimens by semi-quantitative RT-PCR (Figure 6). Consistent with the protein expression, Bax expression was significantly increased in all groups after treatment, and the ACBP-L alone and combinatory treated group exhibited a higher induction (Figure 6b, left panel, Student t test, p < 0.05). Caspase 3 expression was increased in all treated groups, and ACBP-L induced the highest level of Caspase 3 expression (Figure 6b, right panel, Student t-test, p < 0.05). All data strongly suggested that the molecular mechanism of apoptosis was involved by treatments.

## Discussion

In this study, the anticancer effect of ACBP-L in human gastric cancer is demonstrated by suppression of cell line MGC-803 proliferation *in vitro* by MTT assay and morphological observations under light or electron microscope (Figure 1). The IC_50_ of ACBP-L was in the range of 18-28 μg/ml, with a dose and time dependent manner. *In vivo*, ACBP-L exhibited potent anti-tumor effects when used alone, and was able to potentiate Cisplatin chemotherapeutic effect at the lower dose (Figures 2 and 4b). Such combinatory regiment significantly improved the QOL with lower systemic toxicity (Figures 3 and 4a). The tumor specimens harvested from treated groups exhibited increased apoptosis, detected by IHC and RT-PCR, suggesting that the anticancer effect of ACBP-L was due to induced apoptosis through Bax and Caspase mediated mechanisms (Figures 5 and 6).

The novel finding of this study is that ACBP-L alone exhibited potent anti-tumor activity, and additionally, to potentiate Cisplatin chemotherapeutic effects with lower systemic toxicity *in vivo* (Figures 2 and 3). Cisplatin is one of the most broadly used chemotherapeutic agents for treating cancers from the gastric region, the head and neck, and other sites of the aerodigestic tract. However, the high dosing and long-term administration of this medication creates severe systemic toxicity, including gastrointestinal problems, such as intense nausea and vomiting, hair loss, myelosuppression, renal toxicity, and hearing loss. To balance the efficacy of eliminating cancer cells while maintaining the QOL of cancer patients is a great challenge for the oncologist and drug development. Our current study provides a new strategy of utilization of natural existing bioactive peptides, such as ACBP-L used in this study, to potentiate chemotherapeutic effects and minimize toxic effects. In natural resources, broad spectrums of bioactive peptides exhibit regulatory activities that are involved in different biological processes. These natural peptides exist, but in the relatively low amounts or at the low levels of activities. Using immunization protocol with cancer tissue extracts is one way to enrich or activate such naturally exiting peptides. During the process of the isolation and purification of ACBP, we observed significantly higher peaks of these peptides from the induced liver or spleen, when compared with those isolated from normal organs. The enrichment of the recovered peptides is more than three folds after the purification (Su X, unpublished observations). In addition, we compared the anti-tumor activity between the peptides isolated from normal or induced spleens, using the same amount of materials. We observed both normal or induced peptides exhibited anti-tumor proliferation activity *in vitro* using MTT assay. However, a stronger inhibition of tumor cell proliferation, near two-fold increase, was observed in the peptides from induced spleens when compared with the peptides isolated from normal spleens (Su X, unpublished observations). We also compared the peptide biochemical profiles of ACBP isolated from goat spleens [14] or livers, which exhibited similar elution time and characteristics of elution peaks through MPLC (data not shown). Furthermore, ACBP not only exhibited anti-tumor activity of gastric cancer lines MGC803 as shown in this manuscript and SGC-7901 (unpublished data), but also exhibited a broad activity of anti-tumor effects in different cancer types *in vitro* and *in vivo*, regardless immunized cancer types, suggesting it seems not related to specific immune recognition. We have previously published several studies to show the broad anti-tumor activity of ACBP in human myelogenous leukemia line K562 [19], human nasopharyngeal carcinoma line CNE [20], human cholangiocarcinoma cell line QBC939 [21], murine hepatocarcinoma line H22 [22], and human breast cancer line nm231 [23], human colon adenocarcinoma cell line HT29 [24]. However, ACBP showed minimal inhibitory effects on normal cells that we tested, such as human skin fibroblast HS-68 cells and rabbit bone marrow mesenchymal stem cells (Su X, unpublished data). The exact molecular mechanisms of the broad anti-tumor activities and preference against tumor but not normal cells are under investigation. Consistent with the observations *in vitro*, ACBP exhibited minimal side effects *in vivo* (Figure 2). This supports to test ACBP anti-tumor activity in future clinical trials for cancer patients. In addition, one of the advantages for isolating ACBP-L from liver is the higher yield, which is more than 10 times harvest per animal with similar potency than those harvested from spleen (data not shown). The high yield enables us to improve production efficiency and feasibility study of future clinical trials.

In this manuscript, we examined whether ACBP-L could be used as a new adjuvant agent to enhance chemotherapeutic efficacy and reduce toxicity in the treatment of cancer patients. Consistent with the potent anti-cancer effects as previously seen using ACBP from the spleen, in this study, the effects of ACBP-L on the QOL has been demonstrated intuitively by the maintenance of normal body weight when using ACBP-L alone (Figure 3a, b), in contrast to a continuing decreased body weight observed due to the toxic effects of high dosing of Cisplatin alone. Further, the combinatory treated group with lower dosing of Cisplatin exhibited similar anti-tumor effects with a continuing recovery of the body weight that eventually reached normal level (Figure 3a, b). In addition, the QOL was also indicated by average daily food intake, which showed that the amount of food intake in mice treated with high dosing Cisplatin significantly dropped after each treatment. Although, in the combinatory treated group, significant decreased food intakes occurred similarly to the Cisplatin alone group, but the mice recovered quickly and gradually returned to a normal level of food intake. We also observed the suppression of the spleen index in the high dosing of Cisplatin group, but not in the combinatory treated group (Figure 4a). The data suggest that high dosing of Cisplatin could suppress the host immune and defense systems, but the combinatory treated group did not suffer from side effects. Our data strongly support the notion that the combination of chemotherapeutic agents with natural anti-cancer bioactive peptides could be a new strategy for more efficiently treating cancer patients while maintain QOL.

We observed that the viability and metabolism in live tumors exhibited significantly decreased in all three treated groups measured by ^18^F-FDG PET/CT in the live animals (Figure 4b). Integrated PET/CT with ^18^F-FDG is a hybrid of radiation and imaging modalities, which has been recently established in the staging, restaging and therapy response assessment of oncology patients [25]. The machine is capable to determine the glycolytic status in tissues, through an image that shows the tissue distribution of the positron emitter from ^18^F-FDG, a structural analog of glucose. Enhanced glycolysis is one of the most important characteristics of live cancer cells. ^18^F-FDG PET/CT has been proven to be successful as a diagnostic instrument for many solid tumors, including gastric cancer, especially for follow-up after cancer treatment, because it indicates the energy metabolism and viability of the tumor cells [25,26]. In this study, ^18^F-FDG PET/CT method clearly showed consistent results with other biological and molecular measurements, with an advantage of monitoring the anti-tumor effect of different treatments without interrupting the ongoing animal experiment.

Further, ACBP-L induced cell death of tumors also well documented at the cellular level, with morphology of bleb, loss of cell membrane asymmetry and detachment, cell shrinkage, and nuclear condensation (Figure 1b, c). The altered cell morphology is consistent with the molecular mechanism of triggering apoptotic pathways involving BCL and Caspase family members. At the protein level, a significant increase of Bax in ACBP-L alone and in the combinatory groups, and a significantly decreased Bcl-2 in the ACBP-L and Cisplatin groups were observed (Figure 5), suggesting that the mechanisms involved could be slightly different. Consistent with the protein expression, Bax expression at the mRNA level was also strongly induced in the ACBP-L alone and in the combinatory treated groups (Figure 6). The Bcl family, whose members may be antiapoptotic (such as Bcl-2) or proapoptotic (such as Bax), regulates cell death by controlling the mitochondrial membrane permeability during apoptosis [27]. Bax is a 21-kD program partner associated with Bcl-2, and exhibits an extensive amino acid homology with Bcl-2 and forms homo- or heterodimers with Bcl-2 in cells. When BAX predominates, programmed cell death is accelerated, and the death repressor activity of Bcl-2 is countered, such that the ratio of Bcl-2 to BAX determines survival or death following an apoptotic stimulus [28].

While anti-apoptotic effect of Bcl-2 is through the inhibition of the release of Cytochromum C from mitochondria for Caspase activation [29-31], all three treated groups also exhibited an induction of Caspase protein and mRNA expression (Figures 5 and 6). A relatively stronger induction of Caspase 3 was observed in the ACBP-L treated group, while a stronger induction of Caspase 8 was observed in the combinatory treated group (Figure 5). In apoptosis cell, Caspase initiates the opening of the Permeability Transition (PT) aperture of the mitochondria and regulates apoptosis via regulating transmembrane electrochemical gradient [32]. The mechanisms of ACBP-L induced apoptosis are consistent with the apoptotic morphology of altered and destroyed cell surface membrane and structure observed through light and scanning electron microscopes (Figure 1b, c) [33-35].

## Conclusions

We showed that ACBP-L potently inhibited gastric cancer cell proliferation and induced apoptosis *in vitro*. ACBP-L alone exhibited potent anti-cancer effects and potentiated Cisplatin chemotherapeutic effects *in vivo*. ACBP-L alone, or combined with lower dosing of Cisplatin, significantly improved host QOL without compromising therapeutic effects. ACBP-L and combinatory therapy induced cell apoptosis through modulation of BCL and Caspase pathways. Our study suggests that ACBP-L alone, or in combination with chemotherapy agents, could enhance anti-cancer effects and improve patient QOL, which could be a new therapeutic approach for further development against gastric or other neoplasms.

## Material and methods

### Production and purification of ACBP-L from immunized goat liver

The research involved with human subjects were performed following "Ethical Principles for Medical Research Involving Human Subjects". Anonymously primary human gastric tissue samples were obtained from The Affiliated Hospital, Inner Mongolia Medical University, with the approval of the Ethics Committee of Inner Mongolia Medical University, (2012-SWLL-001). All animal experiments were carried out under protocols approved by the Animal Care and Use Committee of the Inner Mongolia Medical University, and were in compliance with the international guidelines (Guide for the Care and Use of Laboratory Animal Resource, National Research Council, USA). ACBP-L was produced and extracted using the following steps. Goats were immunized five times with an interval of one week through a series of injections with human gastric cancer extracts. The livers were harvested from immunized animals. The tissues were subjected to several rounds of ultrasonication (Ultrasonic Disrupter, Model F525 from FLUKO Company, China). After centrifugation at 14,000 rpm for 10 min, the supernatants were collected and ACBP-L was isolated through mesolow preparative liquid chromatography (MPLC, Model YFLC-AI-580 from YAMAZEN corporations, Japan). MPLC is a commonly used protein and peptide purification chromatography, which can separate relatively large amount of materials. The molecular weight of eluted ACBP-L is ~8000 Dalton based on measurement with SDS-PAGE gels [14]. ACBP-L was included in an invention patent of Dr. Su Xiulan’s ACBP-L laboratory and is protected by the Chinese national patent bureau (patent number: ZL96122236.0, and international patent: A61K35/28).

### Cell culture

Gastric adenocarcinoma cell line MGC-803 was kindly provided by Professor Ke Yang (Beijing University, Health Center, Beijing, China). Cells were maintained in RPMI1640 culture medium (Invitrogen, USA), which was supplemented with 10% heat-inactivated fetal bovine serum (FBS, TBD Science, China), 100U/ml penicillin, and100 U/ml streptomycin, and cultured in a humidified atmosphere of 5% CO_2_ at 37°C.

### MTT assay

Cell proliferation was measured by MTT assay [3-(4,5-dimethylthiazol-2-yl)-2,5-diphenyltetrazolium bromide, TBD Science Co., China]. MTT was dissolved in sterile PBS at room temperature, sterilized by passing through a 0.22 μm filter, and stored in the dark at 4˚C. MGC-803 human gastric cells (5×10^3^/well) were placed in 200 μl of culture medium and incubated overnight. After 24 h, cultures were treated with various doses of ACBP-L in triplicates. MTT reagent (20 μl) was added at different time points and then incubated at 37˚C for 4 h. Following vibrating on a shaker for 10 min, the plates were measured for absorbance at 490 nm wavelength using a microtiter plate reader. Drug concentrations that inhibited proliferation by 50% (IC_50_ values) were calculated from dose-response plots by linear regression modeling of the logarithmic form of the equation.

### Scanning electron microscope

MGC-803 cells were cultured in RPMI-1640 medium containing 20 μg/ml of ACBP-L for 48 hrs. All cells were fixed in 2.5% glutaraldehyde in 0.1 M cacodylate buffer (pH 7.2) at 4°C for 1 hr. The samples were rinsed in 0.1 M cacodylate buffer several times, and then dehydrated in graded concentrations of alcohol. The ultrathin sections were dried with Vacuum plating apparatus and treated by spray-gold with ion sputtering equipment (JEOL Company, USA). Then the specimens were examined with a S-3400 N scanning electron microscope (JEOL Company, USA) operated at 15 KV.

### Xenograft tumor model and administration of ACBP-L and Cisplatin

All animal experiments were carried out under the protocol approved by the Animal Care and Use Committee of the Inner Mongolia Medical University, as previously described. Five-week-old athymic nude female mice (BALB/c nu/nu, Institute of Laboratory Animal Sciences, Chinese Academy of Medical Sciences, Beijing, China) were housed in a sterile animal facility and inoculated with MGC-803 cells (2 × 10^7^) in 0.2 ml of PBS subcutaneously. All mice developed single palpable tumors, with the average volume of the tumors equaling ~10 mm^3^ at day 3, following inoculation. The mice were then randomized into four groups, including control, ACBP-L alone, Cisplatin alone, and ACBP-L plus Cisplatin. Each group contained 8 mice (n = 8). The drugs were administered via intraperitoneal injection as follows: control: 0.2 ml of 0.9% NaCl daily for 30 days; ACBP-L alone: 7 μg/mouse ACBP-L in 0.2 ml daily for 30 days (ACBP-L batch#2008-10); Cisplatin alone: 5 mg/kg every 4 days for total 4 doses, at day 6, 11, 16, 21, according to the manufacturer’s protocol (batch# 806027CF, Qilu Medicine Ltd, Jinan, China). The combinatory treatment group of ACBP-L and Cisplatin: 7 μg/mouse ACBP-L in 0.2 ml daily for 30 days plus 5 mg/kg Cisplatin twice every 10 days at day 6 and 16. The mouse body weight, the amount of water and food intake, as well as vital signs and living status, were checked daily. Tumor volume was measured and calculated as follows: the longer diameter, designated as “a”, and the shorter one as “b” of the tumors were measured by Vernier calipers, and the tumor volume (TV) was calculated using the equation: TV = ab^2^/2. The growth curve of tumors was plotted using the mean and standard deviation.

### Measurement of body weight, food intake and collection of tumor specimens

Body weight was measured once every other day, and food intake was measured by weighing the food left in the cage. To calculate the average daily food intake per mouse in a cage, the amount of food present was subtracted from that of the previous day, and then divided by the number of mice in the cage. After euthanasia, tumors, livers, and spleens were collected, weighed, and dissected. The spleen index was calculated by the spleen weight measured in mg versus the mouse body weight measured in grams. Portions of tissues were frozen at -80°C, and portions of tissues were fixed in formalin and embedded in paraffin.

### Positron-emission tomography-computed tomography (PET/CT)

^18^F-deoxyglucose (^18^F-FDG) Positron radioactive tracer was synthesized by medical cyclotron (MINITrace, General Electric Co., Milwaukee, USA) and FX-FN chemosynthesis system (TraceLab, General Electric Co.). Mice were anesthetized by Ether 60 min after tail intravenous injection of 0.2 millicurie (mci) ^18^F-FDG and scanned systemically by 2D positron-emission tomography-computed tomography (PET-CT). Figures from PET-CT were analyzed in Xeleris by two experienced radiologists. The abnormal thick, thin, or defect of radioactivity uptake in inoculation site was excluded. Radioactivity uptake from tumor (Target) was divided by uptake from spine (Non-Target) and presented as Target/Non-Target ratio (T/NT).

### Immunohistochemistry (IHC)

Paraffin embedded tissues were sectioned at a thickness of 4um, and stained with hematoxylin and eosin, and immunohistochemistry (IHC). IHC protocol was modified from S-P Method (Maixin Biological Technology Development Co., Fuzhou, China). Briefly, after deparaffin, antigen retrieval was carried out in a microwave oven for 10 min. The antibodies used were mouse anti-human Bax antibody (90107254D1); mouse anti-human Bcl-2 antibody (90104014 F1), from Maixin Biological Technology Development Co.; rabbit anti-Caspase3 (KGA717, Keygene Biological Technology Development Co., Nanjing, China); and rabbit anti-Caspase 8, (Santa Cruz Biotechnology Co., Santa Cruz, CA, USA). S-P hypersensitive kits (mouse and rabbit, 812059710) and DAB reagents (806180031) were from Maixin Biological Technology Co. Sections were stained with 3, 3-diaminobenzidine (DAB), counterstained with haematoxylin, and dehydrated in xylene, and mounted. The immunohistochemical staining was observed under light microscope (Olympus, Tokyo, Japan), and quantified by Olympus CX41 image analysis system.

### Total RNA extraction and RT-PCR

Total RNA was extracted from tumor specimens using TRIZOL reagent (Invitrogen, USA) according to the manufacturer’s protocol. The quality and concentrations of RNA were measured by a Du-800 UV spectrophotometer (Beckman, USA). Reverse transcription was performed using an RNA PCR Kit (AMV) Version 3.0 (TaKaRa Co., Japan), and the reaction mixture contained: 1 μg RNA sample, 1 μl 10 × buffer, 1 μl dNTP (2 mmol/l), 0.5 μl oligo-dT primer (0.25 μM), AMV RTase 10 U, RNase inhibitor 5 U and DEPC ddH2O up to 10 μl. The mixture was kept at room temperature for 10 min, then was incubated at 42°C for 30 min and at 99°C for 5 min. PCR was performed in 25 μl reaction mixture which consisted of 2 μl reverse transcription products, 5 μl 5X buffer, 0.5 μl each gene specific primers (20 pmol/L), 0.5 μl 10 mM dNTP, 0.75 U Taq DNA polymerase (TaKaRa Co, Japan), with ddH2O added up to 25 μl. PCR cycle parameters were conducted with a pre-amplification denaturation at 94°C for 2 min, followed by 35 cycles of denaturation at 94°C for 30 sec, annealing at 58°C to 65°C for 30 sec, and extension at 72°C for 1 min, with a final extension at 72°C for 5 min. GAPDH was used as an internal control. Amplified PCR products were visualized in a 2% agarose gel electrophoresis containing ethidium bromide. Ratios amplified gene/GAPDH was calculated by software Imagetool 2.0 (University of Texas Health Science Center, San Antonio, Texas, USA). PCR results were confirmed by three repeat amplifications. The sequences of PCR primers were designed using GenBank database and the BLAST program. All primers were synthesized commercially by the TaKaRa Company (Dalian, China), and the experimental condition is presented in Additional file 1: Table S1.

### Data processing and statistic analysis

The calculation of tumor inhibition rate was based on the equation described as follows: Tumor inhibition rate = (tumor weight of control group of saline – tumor weight of medicine group)/tumor weight of control group of saline × 100%.

Data are presented as the mean ± standard deviation (SD). Statistical analysis was performed using *t* test for two groups, *p* <0.05 was considered statistically significant. All statistical analyses were performed using an SPSS program (version 13.0).

## Competing interests

Xiulan Su holds a patent on ACBP-L (Chinese national patent: ZL96122236.0 and international patent: A61K35/28).

## Authors’ contributions

Conceived and designed the experiments: XL S. Performed the experiments: C D, LY S, XM W and HW C. Analyzed the data: JL Z, LY S and Z C. Drafted the manuscript: LY S, JL Z, XL S and Z C. All authors read and approved the final manuscript.

## Supplementary Material

Additional file 1: Table S1 The experimental condition of RT-PCR. Click here for file
