# Supplementary material for: Combination therapy of anti-cancer bioactive peptide with Cisplatin decreases chemotherapy dosing and toxicity to improve the quality of life in xenograft nude mice bearing human gastric cancer
Source: Cell Biosci. 2014 Feb 10;4:7. doi: 10.1186/2045-3701-4-7 (PMC3930002; doi:10.1186/2045-3701-4-7)
Supplement: Additional file 1: Table S1 — The experimental condition of RT-PCR. [file 2045-3701-4-7-S1.doc]

Additional file 1: Table S1 The experimental condition of RT-PCR.

| Primer name | Sequence | Annealing temperature | Cycles | Product length |
| --- | --- | --- | --- | --- |
| Bax F | ACCAAGAAGCTGAGCGAGTGT | 58 ^o^C | 27 | 332bp |
| Bax R | ACAAACATGGTCACGGTCTGC |  |  |  |
| Caspase3 F | CTCGGTCTGGTACAGATGTCGATG | 58 ^o^C | 32 | 540bp |
| Caspase3 R | GGTTAACCCGGGTAAGAATGTGCA |  |  |  |
| GAPDH F | TTACTCCTTGGAGGCCATGTGGGCC | 58 ^o^C | 31 | 465bp |
| GAPDH R | ACTGCCACCCAGAAGACTGTGGATGG |  |  |  |
